# Stromal lymphocytes are associated with upgrade of B3 breast lesions

**DOI:** 10.1186/s13058-024-01857-y

**Published:** 2024-07-08

**Authors:** Tanjina Kader, Elena Provenzano, Madawa W. Jayawardana, Shona Hendry, Jia-Min Pang, Kenneth Elder, David J. Byrne, Lauren Tjoeka, Helen ML. Frazer, Eloise House, Sureshni I. Jayasinghe, Holly Keane, Anand Murugasu, Neeha Rajan, Islam M. Miligy, Michael Toss, Andrew R. Green, Emad A. Rakha, Stephen B. Fox, G. Bruce Mann, Ian G. Campbell, Kylie L. Gorringe

**Affiliations:** 1 Peter MacCallum Cancer Centre, 305 Grattan St, Melbourne, 3000 Australia; 2 The Sir Peter MacCallum Department of Oncology, The University of Melbourne, Parkville, 3010 Australia; 3 Department of Anatomical Pathology, St Vincent’s Hospital, Fitzroy, Australia; 4 Department of Histopathology, Addenbrookes Hospital, and Cambridge NIH Biomedical Research Centre, Cambridge, UK; 5 The Breast Service, The Royal Women’s Hospital, Melbourne, Australia; 6 Edinburgh Breast Unit, Edinburgh, UK; 7 St Vincent’s Breast Screen, St Vincent’s Hospital Melbourne, Melbourne, VIC Australia; 8 BreastScreen Victoria, Parkville, VIC Australia; 9 Department of Clinical Pathology, The University of Melbourne, Parkville, 3010 Australia; 10 Nottingham Breast Cancer Research Centre, Academic Unit for Translational Medical Sciences, School of Medicine, Biodiscovery Institute, Department of Histopathology, University of Nottingham, Nottingham University Hospitals NHS Trust, City Hospital, Nottingham, UK; 11 Histopathology Department, Faculty of Medicine, Menoufia University, Shibin El Kom, Egypt; 12 The Royal Melbourne Hospital, Melbourne, Australia; 13 Department of Surgery, The University of Melbourne, Parkville, 3010 Australia; 14 Laboratory of Systems Pharmacology, Harvard Medical School, Boston, MA 02115 USA; 15 Pathology Department, Hamad Medical Corporation, Doha, Qatar; 16 The Breast Service, The Royal Women’s Hospital, Melbourne, Australia

**Keywords:** Atypical ductal hyperplasia, Lesions of uncertain malignant potential, B3 lesions, Tumour infiltrating lymphocytes, Stromal lymphocytes, Upgrade, Biomarker, Early breast neoplasia, Ductal carcinoma *in situ*

## Abstract

Various histopathological, clinical and imaging parameters have been evaluated to identify a subset of women diagnosed with lesions with uncertain malignant potential (B3 or BIRADS 3/4A lesions) who could safely be observed rather than being treated with surgical excision, with little impact on clinical practice. The primary reason for surgery is to rule out an upgrade to either ductal carcinoma in situ or invasive breast cancer, which occurs in up to 30% of patients. We hypothesised that the stromal immune microenvironment could indicate the presence of carcinoma associated with a ductal B3 lesion and that this could be detected in biopsies by counting lymphocytes as a predictive biomarker for upgrade. A higher number of lymphocytes in the surrounding specialised stroma was observed in upgraded ductal and papillary B3 lesions than non-upgraded (*p* < 0.01, negative binomial model, *n* = 307). We developed a model using lymphocytes combined with age and the type of lesion, which was predictive of upgrade with an area under the curve of 0.82 [95% confidence interval 0.77–0.87]. The model can identify some patients at risk of upgrade with high sensitivity, but with limited specificity. Assessing the tumour microenvironment including stromal lymphocytes may contribute to reducing unnecessary surgeries in the clinic, but additional predictive features are needed.

**Supplementary Information:**

The online version contains supplementary material available at 10.1186/s13058-024-01857-y.

## Introduction

Early detection of malignant breast lesions must be balanced with minimising overtreatment of non-invasive breast conditions in a mammographic screening program [1]. Problematic diagnoses that represent a potential harm of routine mammographic screening are a suite of breast lesions under the general term *lesions of uncertain malignant potential*, also known as B3 lesions [2]. It is estimated that more than 300,000 women are diagnosed with these problematical lesions every year in the United States alone, requiring surgical excision. B3 lesions incorporate precursor lesions as well as those associated with malignancy, including atypical ductal hyperplasia (ADH), breast papillary lesions, flat epithelial atypia (FEA), radial scar and atypical lobular hyperplasia (ALH) [2]. B3 lesions are surgically removed primarily because of the high rate of detecting carcinoma in the excision specimen. Here, we refer to this as “upgrade”, encompassing the scenarios whereby the biopsy missed a nearby carcinoma or there was insufficient indication of invasive breast cancer (IBC) or ductal carcinoma in situ (DCIS) in the core biopsy for a definitive diagnosis. Recent meta-analyses suggest an average 29% risk of upgrade for ADH and 36% for papilloma with ADH [3, 4]. Overall, 17% of all B3 lesions are at risk of upgrade [5], leading to overtreatment of many patients.

Misdiagnosis on biopsy further reduces the effectiveness of mammographic screening. Low-risk atypical lesions such as FEA or columnar cell lesions (CCL) with atypia can be misdiagnosed as ADH [6]. However, if correctly diagnosed, FEA for example exhibits a low upgrade rate and life-time risk of developing IBC [7–9]. The similar architectural features of ADH and low grade (LG) DCIS is another diagnostic challenge, and with only the extent of ducts (2 mm size threshold) as a distinguishing feature, ADH bordering on LG DCIS on biopsy confers a high upgrade rate [10, 11]. In addition, recent meta-analyses showed that ADH can be upgraded to IBC after surgical excision from 9 to 28% of the time [4, 5]. In fact, any B3 lesion with atypia carries a higher risk of missing co-existing malignancy if not excised fully [4, 5]. Thus, such lesions often lead to a recommendation for complete surgical removal [6, 11]. A subset of these tumours can be predicted from mammogram imaging. According to the mammogram imaging category used in this study (Tabar/RANZCR) [12], those with a category 5 (i.e. malignancy) are not problematical lesions in the clinical setting as surgical removal is always indicated. The truly problematical lesions are B3 diagnosed on biopsy with an imaging category 3 (i.e. equivocal) or category 4 (i.e. suspicious for malignancy; for a comparison of BI-RADS with this system, please see the Supplementary Methods (Supplementary Material 2). Since neither imaging information nor biopsy diagnosis are sufficient to exclude the presence of carcinoma, surgical excision or vacuum assisted excision (VAE) remains the standard of care for all B3 lesions suspected to be ADH in particular [2, 13–15]. There is no robust biomarker to predict upgrade of any B3 lesion, reducing the effectiveness of routine screening.

Tumour infiltrating lymphocytes (TILs) have been evaluated as an independent predictive or prognostic biomarker for IBC [16]. Higher TILs positively correlated with favourable prognosis and overall improved survival in triple negative and HER2 positive IBC [17, 18]. Studies on TILs have shed some light on the immune microenvironment of DCIS and benign breast disease, although the utility of TILs as a recurrence biomarker has not been consistent [19–21]. A study evaluating TILs in DCIS from cases fitting criteria for the COMET trial found that a higher number of TILs correlated with the upgrade rate of LG DCIS to HG DCIS [22]. Although TILs have clinical utility in breast cancer in a number of different domains with high reproducibility among pathologists [23], they have not been evaluated to predict upgrade of B3 lesions. Here we investigate and report whether stromal lymphocytes surrounding ductal B3 lesions could be used to predict upgrade.

## Materials and methods

### Cohorts

This study comprises two phases, with case details provided in Additional Table 1.

1. Exploratory: cases ascertained for our previous genetic studies of ADH and papillary lesions [24, 25], which were identified from the Royal Melbourne Hospital (RMH) pathology archives. These were all excision specimens and included pure ADH (*n* = 11) and ADH with synchronous carcinoma (DCIS or IDC, *n* = 20). Data for LG DCIS cases (*n* = 30), intermediate grade (IG) DCIS (*n* = 36) and high grade (HG) DCIS (*n* = 96) were derived from previously published work [19], with new data from an additional 10 LG DCIS cases from the RMH, St Vincent’s Hospital Melbourne, and Peter MacCallum Cancer Centre [24].
2. Biopsy cohort: cases with core needle biopsies available containing B3 lesions were identified from the RMH (1995–2015) and St Vincent’s Hospital (StVs) databases (2000–2016) (*n* = 295), North West Breast Screen (NWBS, 2004–2013) (*n* = 136) and Addenbrookes Hospital, Cambridge, UK (2016–2021) (*n* = 132), with an addition of 3 upgraded cases from the University of Nottingham, UK (Additional Fig. 1). ALH and LCIS were excluded due to their relatively low upgrade rate. Cases were considered upgraded when DCIS/IBC was diagnosed on subsequent excision within a year after the B3 diagnosis. Cases were considered non-upgraded when no DCIS/IBC was reported on subsequent excision within a year after B3 diagnosis. Upgrade for Australian cases was verified through linkage to the Victorian Cancer Registry.

All patients were treated with surgical excision with the exception of 26 cases treated with Vacuum Assisted Excision (VAE). Treatment policy at the hospitals at the time the data was collected was routine excision of all B3 cases, thus there was no comparable control group treated without excision. Patients with any previous history of breast cancer were excluded (Additional Fig. 1). The concordance information between pre and post biopsy (i.e. that the needle targeted the exact area that was identified to be biopsied) was also recorded from Breast Screen Victoria (BSV, available from 160/387 Victorian cases, 41%) and Cambridge. Concordance was determined at the multidisciplinary team meeting (radiologist, surgeon, pathologist) after the biopsy results were returned. Three cases from the Cambridge cohort were excluded based on discordance (Additional Fig. 1).

### Histopathological criteria for ADH, other ductal B3 lesions and papillary lesions

The initial diagnosis of the Australian cases was carried out between 1995 and 2016 by multiple different pathologists. With wider use of immunohistochemical markers, the definition and clinical practice for some of these lesions may have changed since then. Diagnostic hematoxylin and eosin (H&E, 45 cases excluded when not available) stained tissue sections were therefore reviewed by a specialist breast anatomical pathologist (N.R., A.M., P.H., E.P) to ensure consistent diagnoses under current criteria. This review process was blinded to the outcome of the subsequent excision (upgraded or not). The detailed criteria are provided in Additional Information.

Upon re-review, 77 cases were confirmed as ADH in the Australian cohort. Twenty cases were considered LG DCIS on review and were excluded from the cohort. Other cases that did not meet the criteria of ADH were considered to be FEA (*n* = 11), radial scar (*n* = 7) and benign lesions such as CCL and UDH (examples in Additional Fig. 2). FEA and radial scar were still included as B3 lesions, however other cases (*n* = 37) were grouped as “benign ductal lesions”. Only one out of 35 upgraded cases did not meet the criteria for ADH and was described as FEA and radial scar. Most of the H&Es from surgical blocks were unavailable and therefore, were not re-reviewed. If upgraded, the type and grade of DCIS or IBC was recorded from their pathology report at the time of diagnosis.

### Assessing stromal lymphocytes on H&E stained sections

The assessment of stromal lymphocytes was based on H&E sections from the core biopsies of any B3 lesions. All were assessed by T.K. except the Cambridge cohort assessed by E.P. The method to assess stromal lymphocytes of B3 lesions followed that previously published for assessing TILs of DCIS by Hendry et al. [19]. This method was developed by Pruneri et al. [20] for DCIS and other pre-malignant lesions, supported by the recent guidelines of the International Immuno-Oncology Biomarker Working group [23]. The method was also recently utilised for benign breast disease [21]. Since the B3 lesions are not tumour, the term used in this manuscript is stromal lymphocytes instead of TILs.

**Fig. 1:**
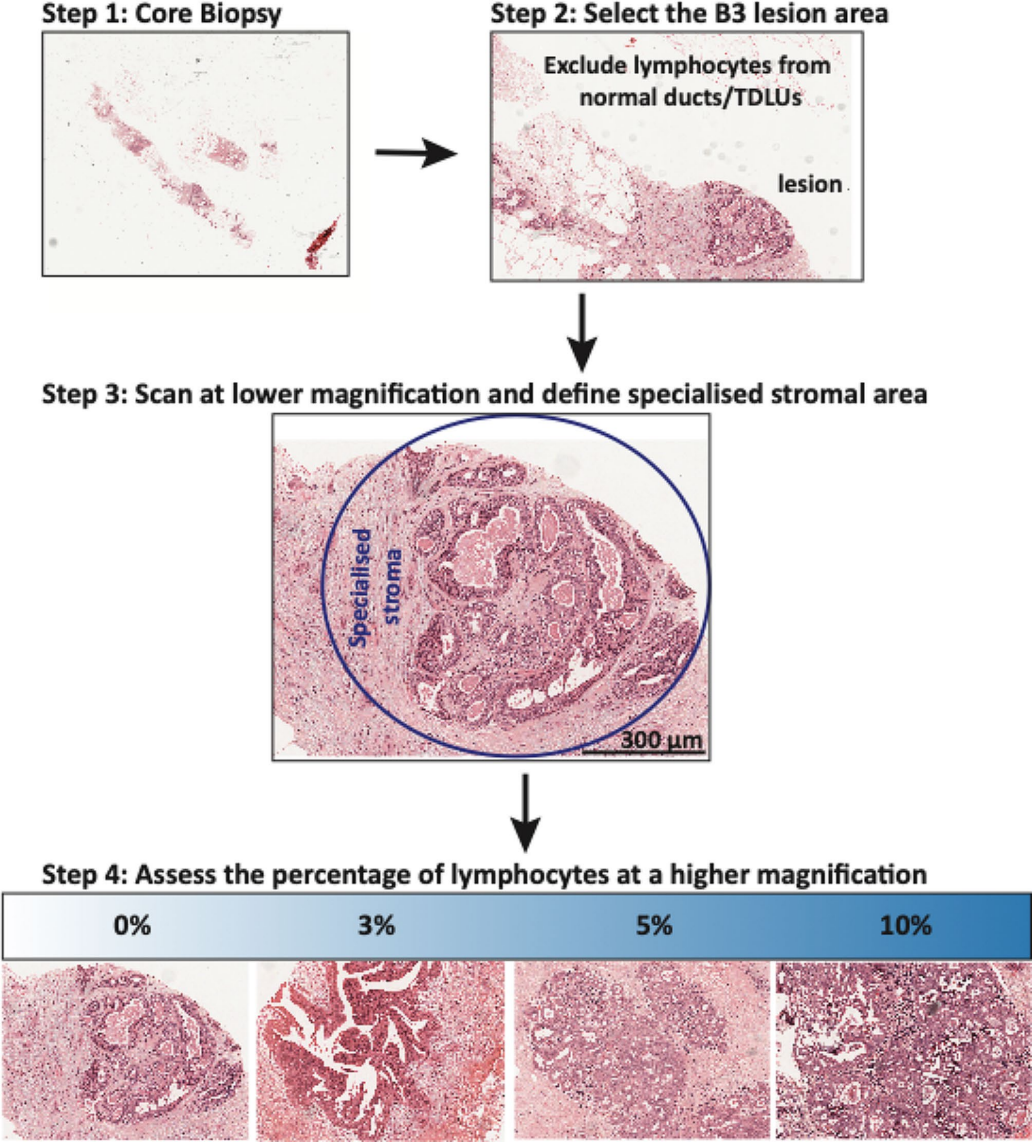
Step wise method for the evaluation of stromal lymphocytes of B3 lesions on H&E biopsy sections. This method of counting TILs from benign breast disease has recently been published by International Immuno-Oncological Working group (www.tilsinbreastcancer.org) [26]

In brief, the stromal lymphocytes of B3 lesions were reported for the stromal compartment (= % stromal lymphocytes). The stromal component was defined as the area within the “specialised” stroma surrounding the B3 lesions (Fig. 1). If the specialised stroma was not clearly visible, the lymphocytes clearly surrounding the B3 lesions were assessed. The specialised stroma was defined as the area surrounding the duct within two high power microscope fields (~ 1 mm). Any minimum, partial; grouped/scattered stromal lymphocytes were taken into account. Stromal lymphocytes of any normal ducts or terminal-ductal lobular unit (TDLUs), if present, were noted while assessing, however, were not taken into account for the total stromal lymphocytes according to the International Immuno-Oncology Biomarker Working Group guidelines [23]. In biopsies with a mix of lesions, such as ADH and FEA, the stromal lymphocytes were counted across all lesions and an average score was used. For biopsies with multiple blocks per case, all blocks were assessed and an average lymphocyte count was used. Cases were excluded from stromal lymphocyte assessment if there was a lack of stromal components (< 1 mm) surrounding the ducts of the lesions (Additional Fig. 3). This assessment was extremely crucial since many IDP in particular lacked the stromal component on biopsies. The total number of assessed cases were ADH (*n* = 108, 47 upgraded), other non-ADH ductal B3 lesions (i.e. FEA, radial scar; *n* = 33, 5 upgraded), non-B3 lesions (CCL, UDH; *n* = 37, none upgraded), IDP (benign and with ADH) (*n* = 166, 26 upgraded) (Additional Fig. 1).

### Ethics approval

This study was conducted under ethical approval from the Peter MacCallum Cancer Centre (HREC #12–64), (HREC#19/194), Melbourne Health (HREC# 2012.119), (HREC#2019.390), St Vincent’s Hospital (HREC #022/19), the North West-Greater Manchester Central Research Ethics Committee 15/NW/0685 and MTA transfer agreement between PMCC (HREC#19/194) and Cambridge University Hospitals NHS Foundation Trust Hills Road Cambridge.

### Statistical analysis

Differences between those in the upgraded vs. non-upgraded groups were assessed using the Mann-Whitney U test for continuous variables, chi-squared test for categorical variables, ANOVA for certain comparisons, and negative binomial model for count variables. A multivariable logistic regression model with patient age (non-linear), type of lesion (ADH, FEA/Radial Scar, benign IDP and IDP + atypia) and lymphocytes as a continuous variable was fitted to predict the binary outcome of upgrade status for the combined cohort. Four cases had missing age data. We measure the model discrimination ability using the area under the curve (AUC) [27, 28] (a.k.a. concordance (C) statistic in logistic models) with its 95% confidence intervals and discrimination slope [29]. Models were assessed for overfitting using shrinkage estimates and internally validated via bootstrap sampling [27, 28]. A nomogram was constructed based on the multivariable logistic model. R (v.4.3.1) [30] and RStudio (v 2023.09.1, Posit Software) were used to generate graphs and perform statistical analysis. A p-value of < 0.05 was considered significant.

## Results

**Fig. 2:**
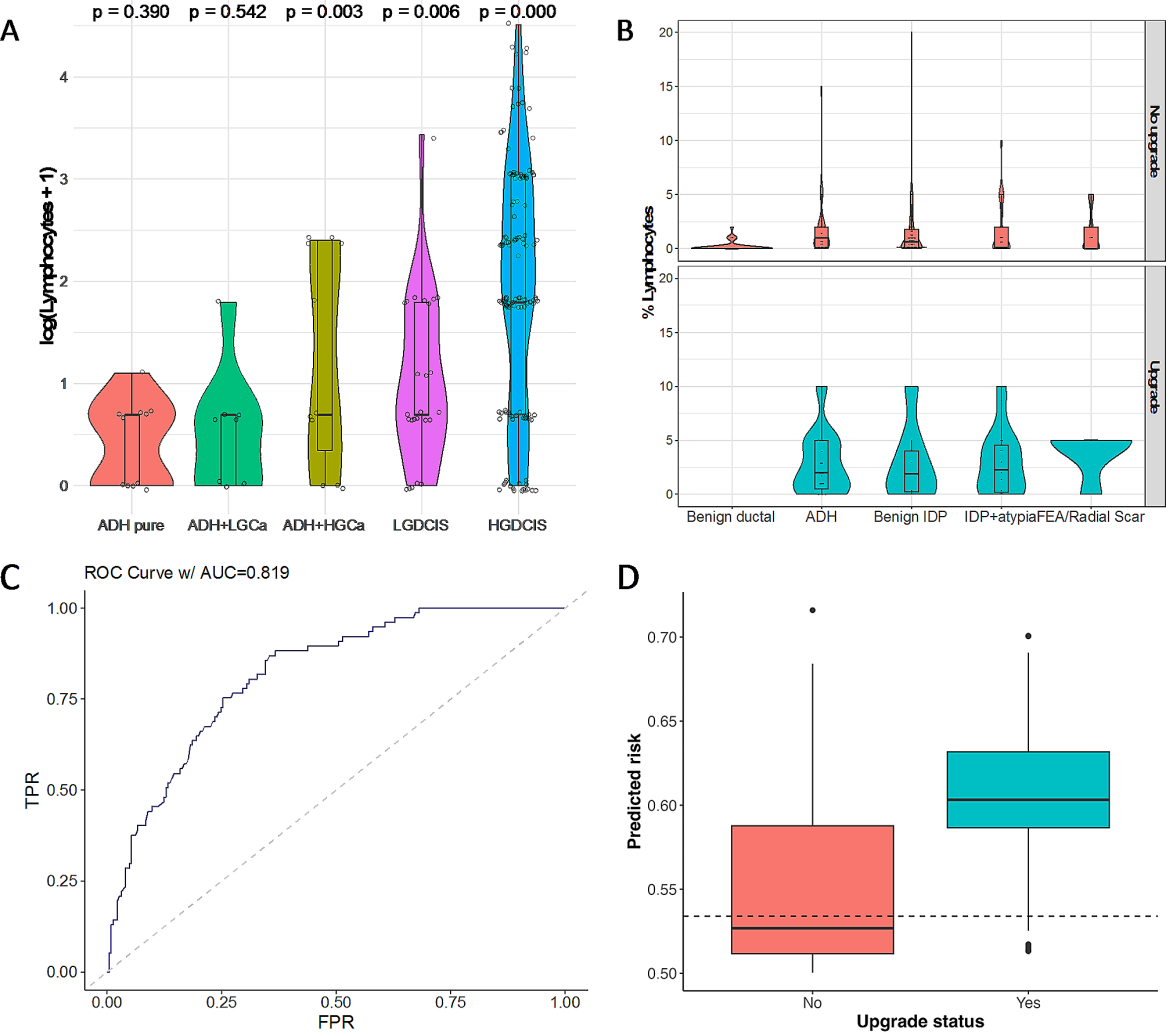
Lymphocytes have predictive value for upgrade. **A**. ADH synchronous with HG carcinoma (Ca) has higher stromal lymphocytes than pure ADH or ADH with LG carcinoma (note log scale). The lymphocyte counts were also compared to previously published DCIS cases [31]. P-values from negative binomial model. Internal box-plot represents median and quartiles; circles are individual cases. **B**. B3 lesions on biopsy (ADH, papillary and FEA/Radial scar) have more lymphocytes than benign ductal lesions, and this is further elevated in the context of upgrade (no benign lesions upgraded). **C**. ROC curve of model to predict upgrade with age (non-linear), type of lesion and percentage lymphocytes as a continuous variable. **D**. Box plot of predicted probabilities of non-upgrade and upgrade (Brier score = 0.145) using the fitted logistic regression model. This shows the difference in predictions between the outcomes. A better discriminating model will show less overlap between those with and those without the outcome. The dashed line indicates a probability threshold corresponding to a 10% false negative rate

We previously conducted genetic studies of ADH and papillary lesions, both pure and in the context of coexisting carcinoma (DCIS or IDC) [24, 25]. In these excision specimens, we observed that pure ADH seldom had many stromal lymphocytes (median 1%, range 0–2%), but that there were often lymphocytes in the stroma surrounding ADH coexisting with HG carcinoma, but not LG carcinoma (Fig. 2A), particularly if the ADH was in close proximity to the carcinoma (two-way ANOVA, Grade = 0.04, Block = 0.06, Additional Fig. 4). Pure DCIS showed a significant increase in lymphocytes compared to pure ADH (*p* = 0.006 vs. LG DCIS, *p* < 0.0001 vs. IG-HG DCIS, negative binomial model) (Fig. 2A) with medians of 1% (range 0–30%) and 5% (range 0–90%), for ADH and DCIS respectively.

We hypothesised that in a core biopsy with a lesion of uncertain malignant potential (B3), the presence of lymphocytes might suggest a nearby carcinoma that perhaps was not sampled fully (i.e. upgrade), particularly if high-grade, which would be of most concern.

We investigated the stromal lymphocytes in our cohort of B3 lesions including ADH, other non-ADH ductal B3 lesions (FEA and radial scar) and papillary lesions. For comparison, we included benign lesions that had been initially diagnosed as ADH but on modern review were no longer classed as B3 (e.g. UDH). Most of these patients were diagnosed after routine mammogram imaging and were classified as category 3 (indeterminate/equivocal findings), regardless of the upgrade status (Table 1).

**Table 1:** Sample cohort

	Number	Median age (range)^1^	Imaging category 2/3 (%)	Imaging category 4 (%)^2^
ADH	61	54 (41–75)	35 (79.5)	9 (20.5)
ADH upgrade	47	58 (40–79)	22 (75.9)	7 (24.1)
FEA/Radial Scar	28	52 (42–70)	18 (85.7)	3 (14.3)
FEA/Radial Scar upgrade	5	61 (52–67)	5 (100)	0 (0)
Benign IDP	109	53 (23–81)	79 (81.4)	18 (18.6)
Benign IDP upgrade	7	59.5 (46–72)	5 (83.3)	1 (16.7)
IDP + atypia	31	59 (28–84)	14 (70)	6 (30)
IDP + atypia upgrade	19	58 (36–77)	14 (82.4)	3 (17.6)
Benign ductal	37	55 (31–72)	9 (69.2)	4 (30.8)

(1) P value = 0.004 for all upgraded B3 vs. not upgraded B3 (excluding benign ductal); two-sided t-test. (2) P value = 1 for all upgraded B3 vs. not upgraded, combining category 2 and 3 and comparing to category 4; Chi-squared test

The presence of lymphocytes in non-upgraded ADH cases ranged from a score of 0–15%, with a median of 1% (Fig. 2B, Additional File 1). This level was higher than the presence of stromal lymphocytes in benign lesions and radial scar (both 0–2%, with a median of 0% (Fig. 2B)) but similar to FEA (median 1%, range 0–5%).

The upgraded ductal B3 cases were mostly diagnosed on biopsy as ADH (*n* = 47), except for four cases of FEA and one case of FEA with radial scar. The upgrade tumours spanned a range of grades and histological types (Additional File 1, Additional Fig. 5). Overall, 34.6% (18/52) of upgraded ductal B3 cases were upgraded to LG DCIS, and 17% to G1 IBC (9/52). IG DCIS comprised 17% (9/52) and G2 IBC 5.8% (3/52) with the remainder HG DCIS (9/52, 17%), lobular carcinoma and DCIS of unspecified grade. The presence of stromal lymphocytes in the biopsy section of upgraded ADH cases ranged from a score of 0–10%, with a median of 2% (Fig. 2B, Additional File 1), which was significantly higher than non-upgraded ADH (*p* = 0.017, Mann-Whitney U-test) and benign lesions (*p* < 0.0001) (Fig. 2B). Upgraded FEA and radial scar also had elevated lymphocytes, with a median of 5% (range 0–5%).

We also investigated core biopsies with benign and atypical papillary lesions. It is noteworthy that unlike ADH/ductal B3, papillary lesions frequently did not meet the criteria to count lymphocytes (44% (66/150) of biopsies, Additional Fig. 1). There was a significant difference in total lymphocytes between non-upgraded papillary lesions and upgraded (*p* = 0.007, Mann-Whitney U-test), although this difference was stronger in the papillomas with atypia. Benign papillomas had a median of 0.6% (range 0–20%) in non-upgraded biopsies vs. 1.9% (range 0–10%) in upgraded biopsies (*p* = 0.21, Mann-Whitney U-test). IDP with atypia was similar, with a median of 0.1% for non-upgraded (range 0–10%) and 2.3% for upgraded cases (range 0–10%, *p* = 0.04, Mann-Whitney U-test). Upgrades were to LG DCIS (*n* = 11, 42%), IG DCIS (*n* = 5, 19%), G1 IDC (*n* = 4, 15%), HG DCIS (*n* = 2), papillary carcinoma (*n* = 2), and DCIS (no grade available, *n* = 2).

Our initial observation suggested that lymphocytes would be more prevalent in biopsies upgraded to high-grade carcinoma. However, there was no significant correlation between the percentage of stromal lymphocytes in biopsies with upgrade and the grade of DCIS/IBC diagnosed on surgical excision (*p* = 0.47, one-way ANOVA, Additional Fig. 4). For example, of the 29 biopsies upgraded to LG DCIS, the median lymphocytes was 2% (range 0–10%) while for those upgraded to HG DCIS it was 2.3% (*n* = 11, range 0–5%).

Degnim et al. [32] showed that patient age was a risk factor for subsequent cancer after a diagnosis of atypical hyperplasia. Therefore, for application in a clinical setting, we developed a logistic regression model with patient age (non-linear), type of lesion (ADH, FEA/Radial scar, benign IDP and IDP with atypia) and lymphocyte count as variables, with an AUC of 0.82 (95% CI: 0.77–0.87, Fig. 2C). This model was sensitive, but had limited specificity: for example, if we accept a false negative rate of 10%, we would have 122/226 (54%) non-upgrades predicted to be upgraded (Fig. 2D). A nomogram is provided in Additional Fig. 6.

For potential clinical application, we attempted to select a threshold for “high” lymphocytes based on the distribution in the non-upgraded compared to upgraded samples (Additional Fig. 7). Although there was no clear-cut threshold, we selected 5% as a potential cut-off for clinical utility. The reproducibility of this threshold was tested in 40 randomly selected cases between three observers 1 (T.K., S.H. and J.M.P.). Assessors were blinded to the outcome (upgraded/non-upgraded). The cases were called either as < 5% or ≥ 5% stromal lymphocytes and this designation was 100% concordant among the observers. However, when re-testing our model with this threshold, it was not as strongly predictive (C = 0.803 compared to C = 0.819), although still better than age and lesion type alone (C = 0.775). Of cases with < 5% lymphocytes, 51/252 (20%) were upgraded, compared to 27/55 (49%) of cases with ≥ 5% lymphocytes.

## Discussion

Many efforts to identify predictive biomarkers of upgraded B3 lesions have failed because of either a lack of sensitivity: the “low risk” group still has a considerable risk of upgrade [33], the predictive feature is not reproducible [34, 35] or the feature is only prognostic after full excision [32, 36]. Overall, predictive features are inconsistent across studies and many of the features require highly experienced pathologists and radiologists to interpret the available data. A risk score was recently developed by Lustig et al. [37] to predict upgraded ADH after using a logistic regression model to select variables. However, the score was based on 5 variables, such as size of multi-modal imaging and the presence of “DCIS-like” or > 1 high risk lesions on core biopsies. Our study on the other hand showed a high sensitivity of predicting any B3 lesions using simpler variables with a high reproducibility. The multivariable logistic regression model using lymphocyte count, patient age and type of lesion as variables reported an AUC of 0.82 in our cohort.

Although we had excellent concordance of calling < 5% and ≥ 5% stromal lymphocytes on cases scored by two experienced breast cancer pathologists, there is concern that this measure could vary in reproducibility. This issue would need to be addressed by a concordance study including non-specialist or less experienced pathologists, particularly given that we could not select a suitable threshold and the nomogram developed used the individual sample percentage value, which could vary between scorers. However, there are now excellent training resources for lymphocyte counting, and reproducibility could be assisted by automated counting using digitized pathology images or the development of a machine learning method [38]. Such an approach would facilitate validation of lymphocytes as a biomarker by increasing the sample size and potentially selecting an optimized threshold.

Cases that are histologically B3 on biopsy but with a mammographic imaging category indicating malignant findings are not problematical clinically due to the clear clinical guidelines for these cases to be surgically excised. On the other hand, B3 cases on biopsy with mammographic findings equivocal/indeterminate or suspicious for malignancy cannot be ruled out for co-existing cancer only based on imaging, leading to potentially unnecessary surgeries. Most of our B3 biopsies were recorded as indeterminate (category 3) on mammogram imaging. Here we developed a predictive marker for ductal B3 lesions based on total stromal lymphocytes, patient age and lesion type that would be cost effective anywhere in the world. Patients with high lymphocytes should continue to have removal of the lesion. In contrast, absence of lymphocytes indicates the B3 lesion could be more likely to be benign. Young patients in this scenario could consider surveillance over immediate surgery, reducing unnecessary surgeries. Increasing use of VAE may reduce the concerns over the impact of removal of all concerning lesions, however, a recent UK audit found that in women eligible for and who underwent VAE, 25.3% and 11.7% of B3 lesions with and without atypia respectively were upgraded [39]. A low level of lymphocytes in a lesion could potentially be a reassuring factor in electing VAE over surgical excision, as the uptake in the 2019 audit was 65–74% of eligible cases. In addition, current UK guidelines recommend surgical biopsy for papillary lesions with atypia: our findings could indicate that such lesions with no lymphocytes could be managed with a less invasive procedure like VAE as the upgrade rate was lower (7/25, 28% vs. 12/25 48%).

Using lymphocytes alone as a marker, without including age and lesion type, would likely lead to extensive under-diagnosis of upgrade (23/78 upgrade cases had no lymphocytes). Nonetheless it was notable that 9/23 upgrades with no lymphocytes were LG DCIS, which potentially are lesions with low risk of progression to more serious pathology if left untreated until the next routine screen. It is important to keep in mind that the distinction between ADH and LG DCIS is often only based on the size of the lesion. Thus, LG DCIS may not be diagnosed at biopsy due to insufficient sampling of the lesion leading to a preliminary diagnosis of ADH. Therefore, it is debatable whether these cases should be considered “upgrades”. Nonetheless, the clinical consequence of a diagnosis of LG DCIS is different from that of ADH, although this may change should surveillance and not surgery become acceptable for LG DCIS. In this study, we included all patients even when upgraded to LG DCIS, because any predictive biomarker to enable safe observation would currently be desirable.

A further limitation is that the ability to apply this method to papilloma biopsies was hampered, with not quite half of the biopsies carrying sufficient stroma to score. There was little difference between the drop-out rate for non-upgrades (42/116 (36%)) and upgrades (15/32 (47%), chi-squared test *p* = 0.37). The drop-out rate for ductal lesions was considerably lower, at 7.4%. There did not appear to be any bias in the cases that dropped out from lack of stroma in terms of upgrade for ADH cases with 1/31 upgraded and 2/32 non-upgraded cases being removed for this reason. The high upgrade rate for ADH in our cohort (44%) could be due to the rigorous pathology review, which reclassified as benign many cases originally called as ADH, none of which were upgraded.

A potential source of bias is the lack of data from patients who did not have removal of the lesion. However, due to clinical guidelines in the UK and Australia, this number is expected to be very small. Finally, despite accessing cases from multiple sources, the sample size in terms of number of upgrades remains relatively small and our model will need to be validated in other cohorts, preferably prospectively.

A few key individual immune cell types were previously investigated in a large cohort of benign breast disease including ADH [40], however, this hasn’t yet been explored for upgrades. The biology of a higher number of lymphocytes and individual immune cells of upgraded cases are yet to be explored, but could reflect the altered microenvironment caused by the presence of nearby carcinoma. The relatively low specificity of high lymphocytes for predicting upgrade could be due to an inability to detect different functional immune cell types by H&E that could be resolved if an as yet unknown IHC marker were added. The sensitivity limitations of stromal lymphocytes for detecting upgrade could reflect the inter-tumoral heterogeneity in TIL presence (some carcinomas have very low TILs) and also intra-tumoral heterogeneity. The biopsy taken could have missed an area of the tumour field with higher lymphocytes. This possibility could be explored through a comparison of the biopsy with the excised carcinoma, which unfortunately was not available for our cohorts. The unavailability of the excision specimens also precluded verification of the excision diagnosis, particularly carcinoma grade, which is an additional limitation of the study.

The strength of our study is extensive pathological review by experienced pathologists to reconfirm the diagnosis of types of B3 lesions. To our knowledge, this is the largest cohort to date analysed for lymphocytes of B3 lesions. This method of counting lymphocytes (or TILs for IBC and DCIS) is already accepted as part of the routine diagnostic procedure at many hospitals worldwide. The implementation of TILs has been informative for prognosis of IBC patients. Here we showed evidence of stromal lymphocytes of B3 biopsies combined with patient age and lesion type as being predictive of upgraded cases, which are a clinical problem that must be addressed before de-escalating treatment for B3 lesions.

### Electronic supplementary material

Below is the link to the electronic supplementary material.

Supplementary Material 1

Supplementary Material 2

Supplementary Material 3

## Data Availability

No datasets were generated or analysed during the current study.
