# Supplementary material for: Stromal lymphocytes are associated with upgrade of B3 breast lesions
Source: Breast Cancer Res. 2024 Jul 8;26:115. doi: 10.1186/s13058-024-01857-y (PMC11232297; doi:10.1186/s13058-024-01857-y)
Supplement: Supplementary file 2 — Supplementary Material 2 [file 13058_2024_1857_MOESM2_ESM.pdf]

Supplementary Methods

Criteria for ADH: The criteria for ADH was according to Page *et al*. [26] and Lakhani *et al.* [27] as previously published [24]: "ADH is characterised by a proliferation within TDLU of a monomorphic population of epithelial cells with generally rounded nuclei that are evenly spaced and have well defined cell borders. The cells form "punched out" (cribriform-like) secondary lumens and/or micropapillae. The cells may grow in arcades or rigid bridges of uniform thickness". The neoplastic cells comprising the proliferation are cytokeratin, CK5/6 negative (surrounding myoepithelial cells show staining for CK5/6) as well as strong and uniform positively stained nuclei for ER as previously published [24]. In distinguishing ADH from LG DCIS, the latter required complete involvement of >2 ducts or partial involvement of ducts >2 mm in extent, in keeping with the criteria described in Lakhani *et al.* [27]. Exclusion criteria of ADH were absence of atypical cells (i.e. UDH), CCL with UDH, and other early neoplasia with atypia such as FEA, radial scar or apocrine hyperplasia. All of these lesions lack the secondary structure of cribriform and/or micropapillary structures, and had only cytological atypia with an architectural structure of CCL or FEA [2, 27] (Supplementary Figure 3).

Criteria for papillary lesions: The criteria followed for benign papilloma or papilloma with ADH were according to Page *et al.* combined with the recent recommendation of the World Health Organisation (papilloma with <3 mm extent of ADH) [27, 28]. p63 and CK5/6 immunohistochemistry (IHC) were evaluated when available to determine the differential diagnosis between benign papilloma and papillary carcinoma (p63+ve = benign papilloma, p63-ve = carcinoma) as well as reconfirming the atypical populations (<3 mm CK5/6 –ve = papilloma with ADH; >3 mm CK5/6-ve = papilloma with DCIS), respectively as previously published [25].

Supplementary Table: Comparison of breast lesion imaging classification methods. Source: https://www.ranzcr.com/college/document-library/breast-imaging-grading-comparison-and-lesion-classification

**BI-RADS**	**BI-RADS descriptor (5th edition)**	**Tabar/RANZCR**	**Tabar/RANZCR descriptor**
BIRADS 0	Incomplete		
BIRADS 1	Negative	Grade 1	Normal
BIRADS 2	Benign	Grade 2	Benign
BIRADS 3	Probably benign	Grade 3	Indeterminate
BIRADS 4A	Low suspicion	Grade 3	Indeterminate
BIRADS 4B	Moderate suspicion	Grade 4	Suspicious
BIRADS 4C	High suspicion	Grade 4	Suspicious
BIRADS 5	Highly suggestive of malignancy	Grade 5	Highly suspicious
BIRADS 6	Biopsy-proven malignancy		
