# Supplementary material for: Stromal lymphocytes are associated with upgrade of B3 breast lesions
Source: Breast Cancer Res. 2024 Jul 8;26:115. doi: 10.1186/s13058-024-01857-y (PMC11232297; doi:10.1186/s13058-024-01857-y)

Supplementary Figure 1. Case inclusion and exclusion

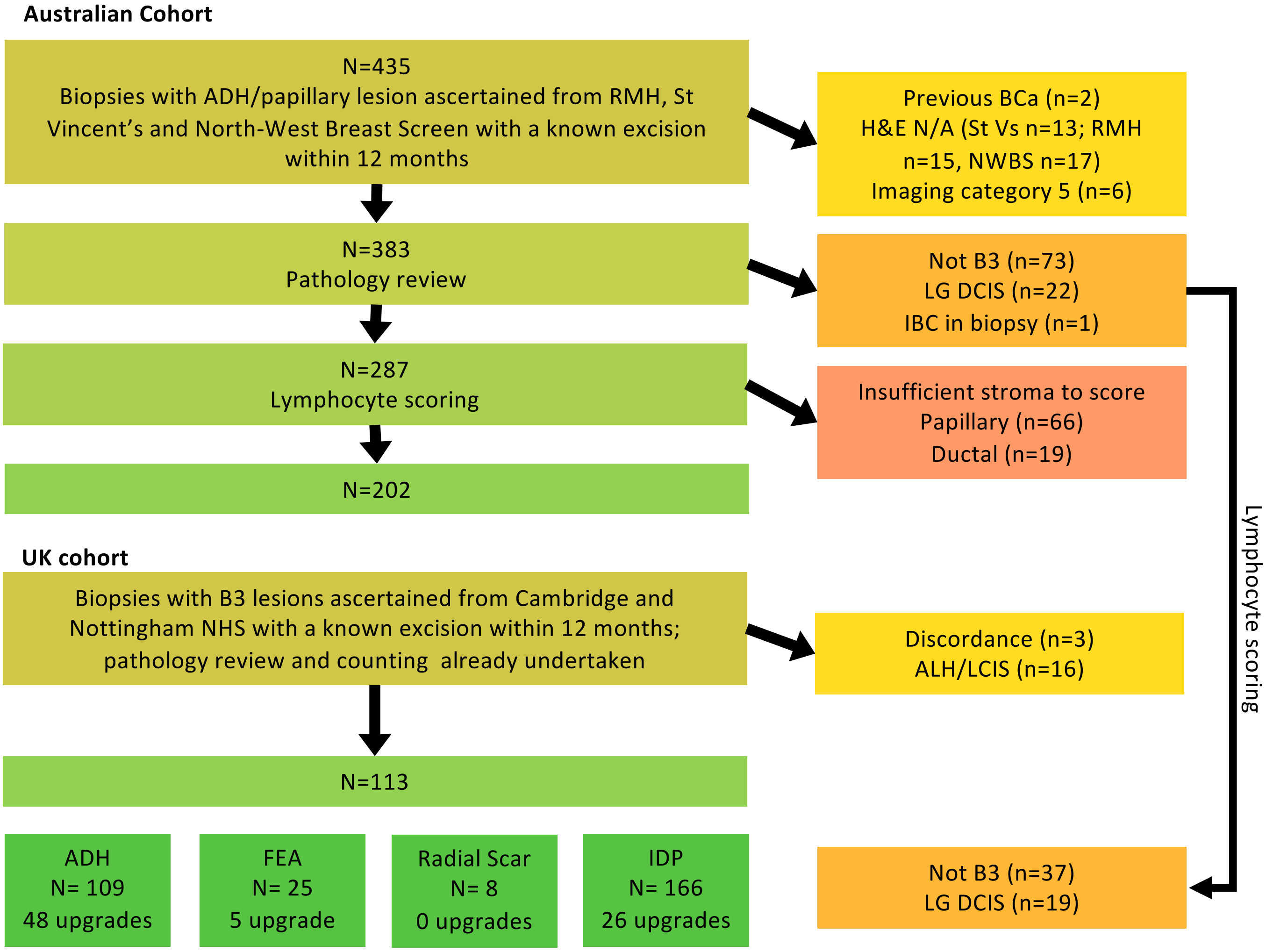

Supplementary Figure 2. Examples of other ductal lesions that were not confirmed as ADH. These ductal lesions include radial scar, columnar cell change (CCL), flat epithelial atypia (FEA) or usual ductal hyperplasia (UDH).

Case SV36: Radial Scar

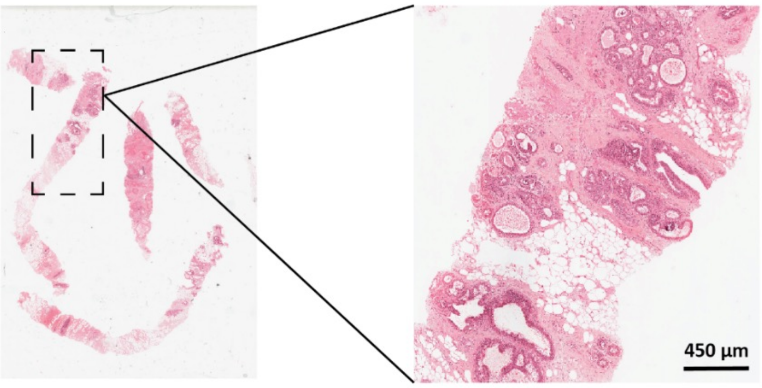

Case SV32: Columnar cell change (CCL), usual ductal hyperplasia (UDH)

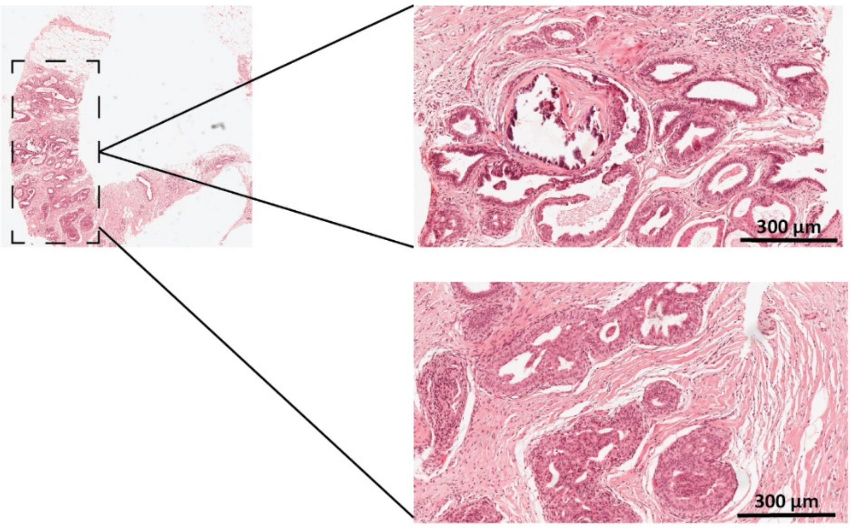

Case SV73: Flat epithelial atypia (FEA)

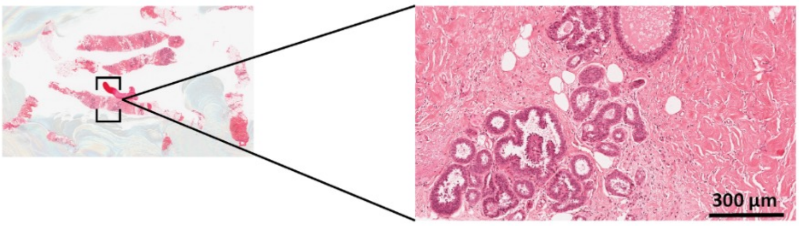

Supplementary Figure 3. Examples of excluded cases from analysis due to lack of specialised stroma in biopsies.

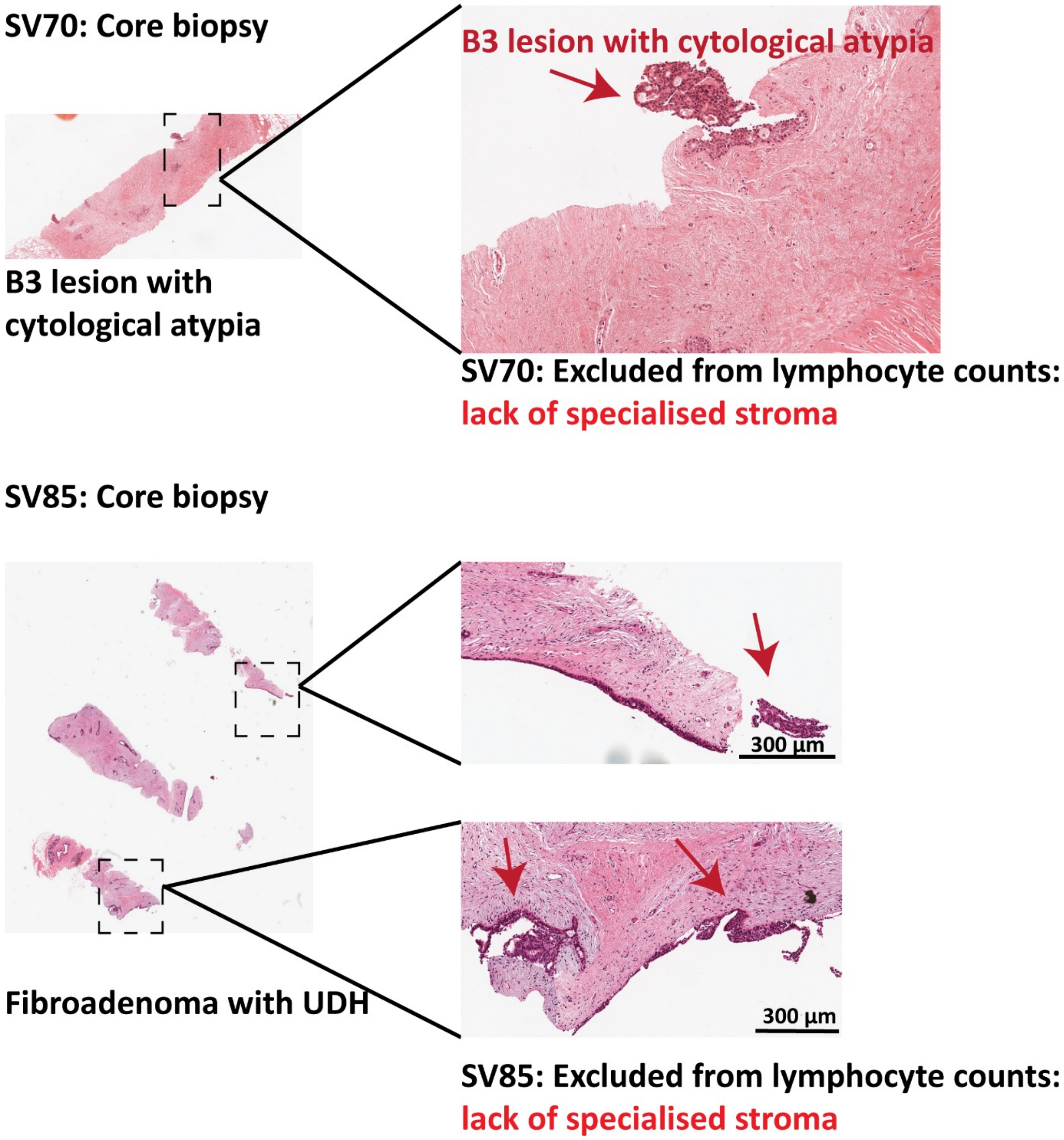

Supplementary Figure 4. Grade of carcinoma and lymphocyte associations. **A**. ADH synchronous with high grade carcinoma is more likely to have high lymphocytes when found in the same block. **B**. Breakdown of grade of upgrade related to lymphocytes

A

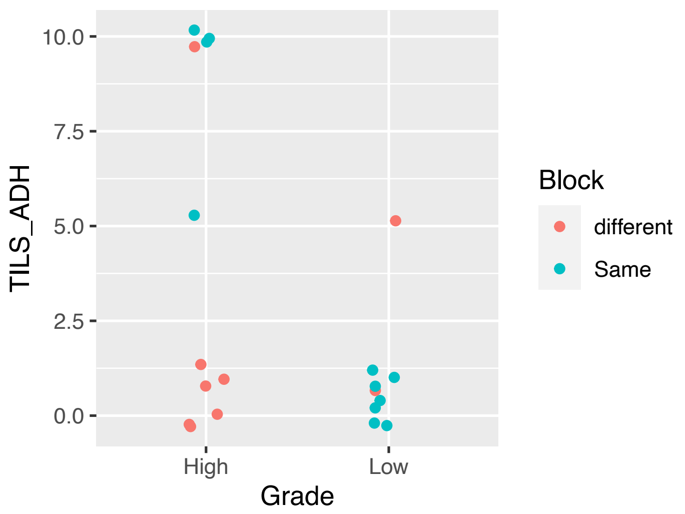

B

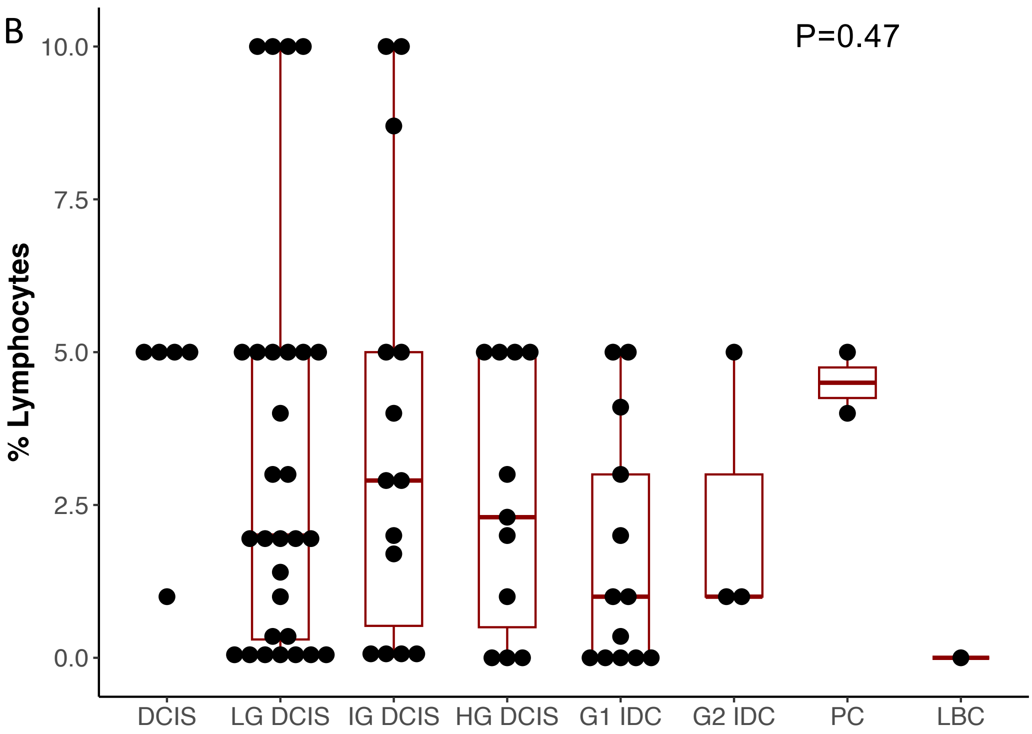

Supplementary Figure 5. Sankey plot showing type of lesion in the biopsy at left, with the outcome of the excision at right

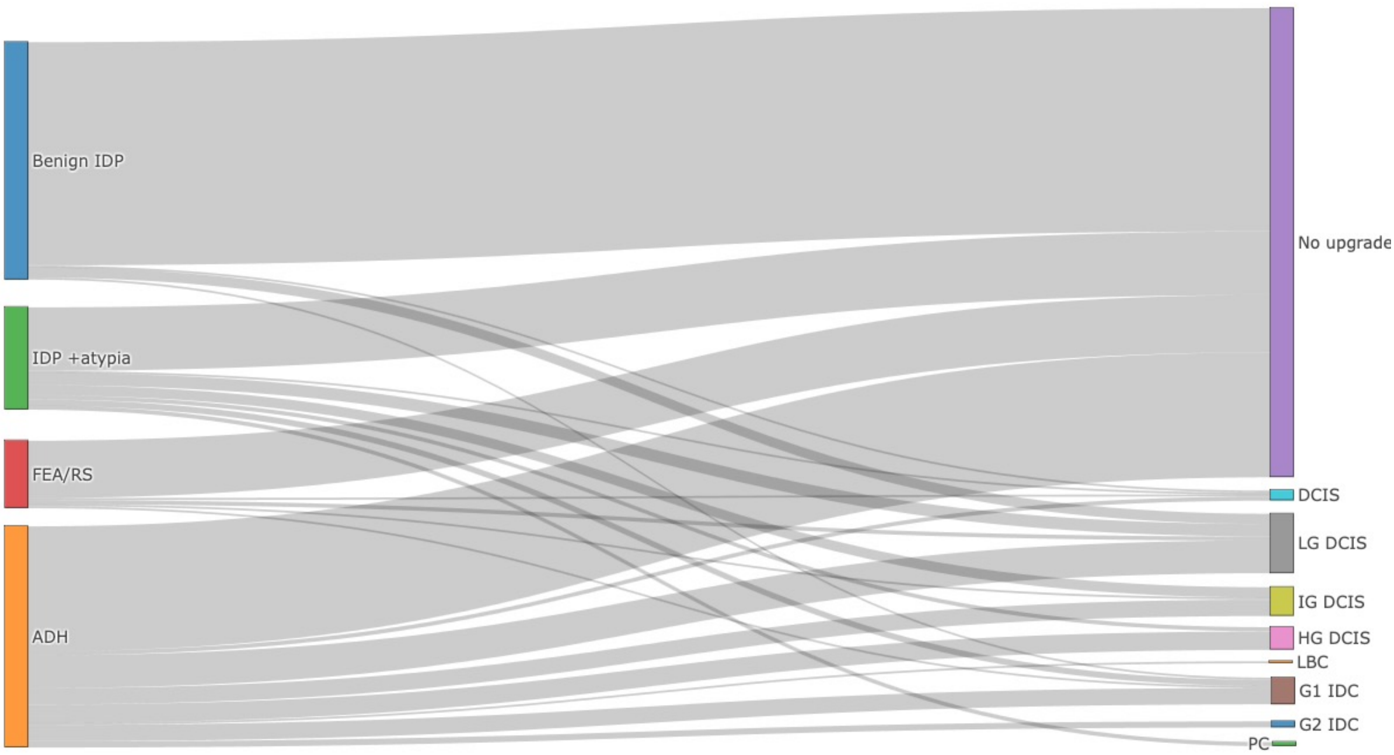

Supplementary Figure 6. A. Nomogram for model to predict upgrad**e. B.** Internal calibration plot of model

A

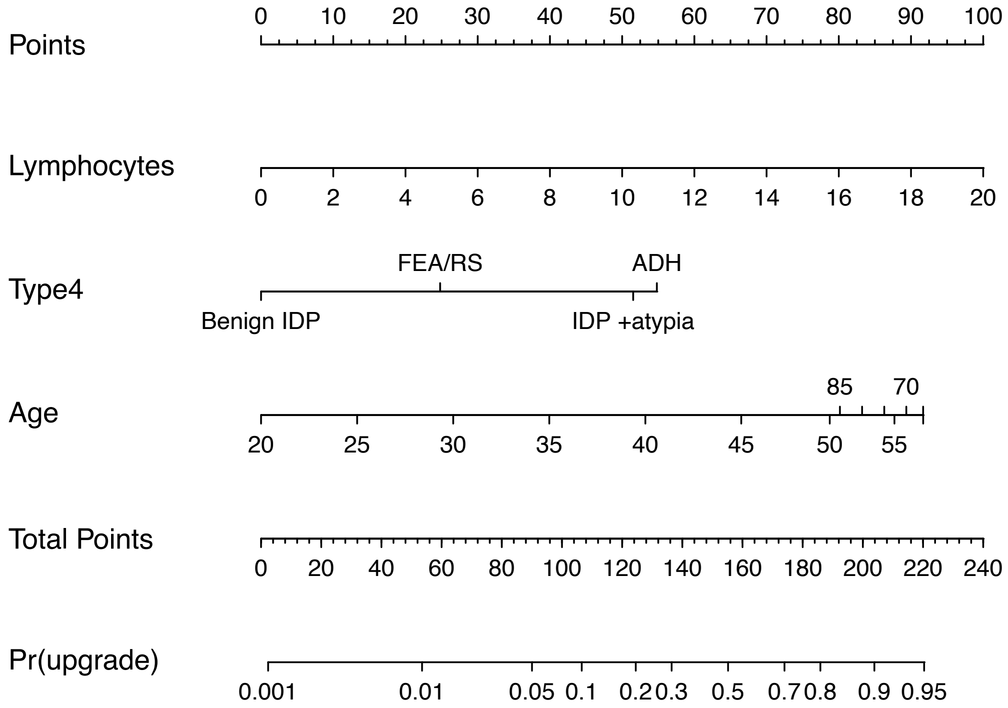

B

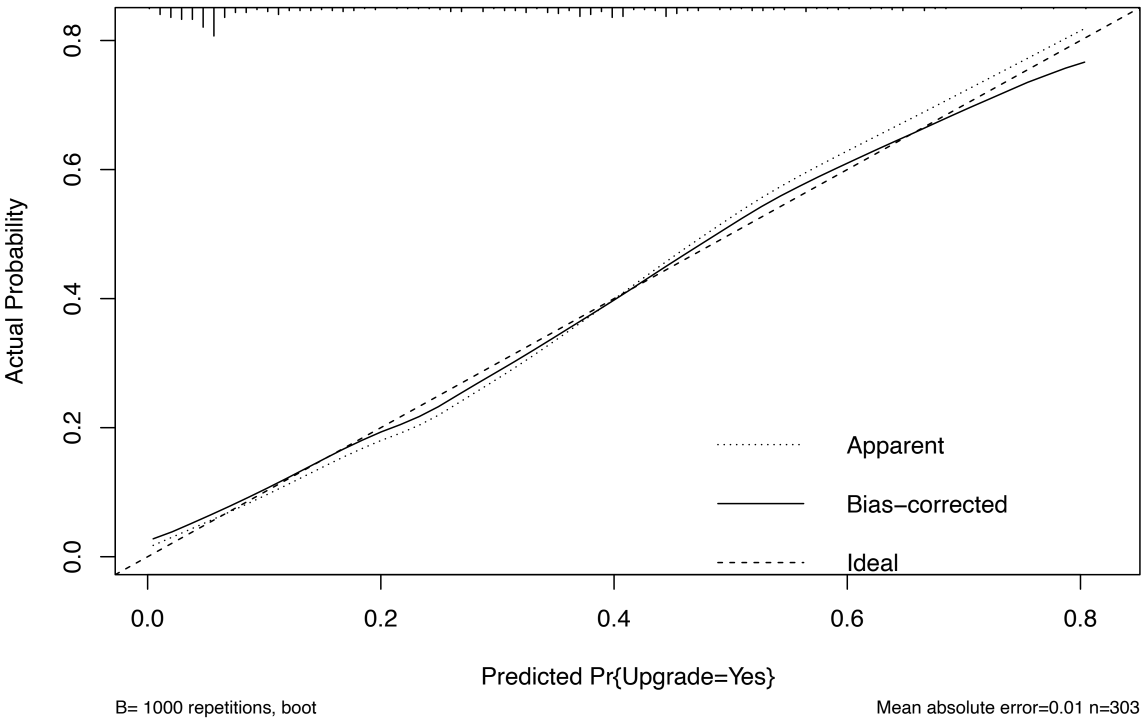

Supplementary Figure 7. Selection of a threshold for lymphocytes

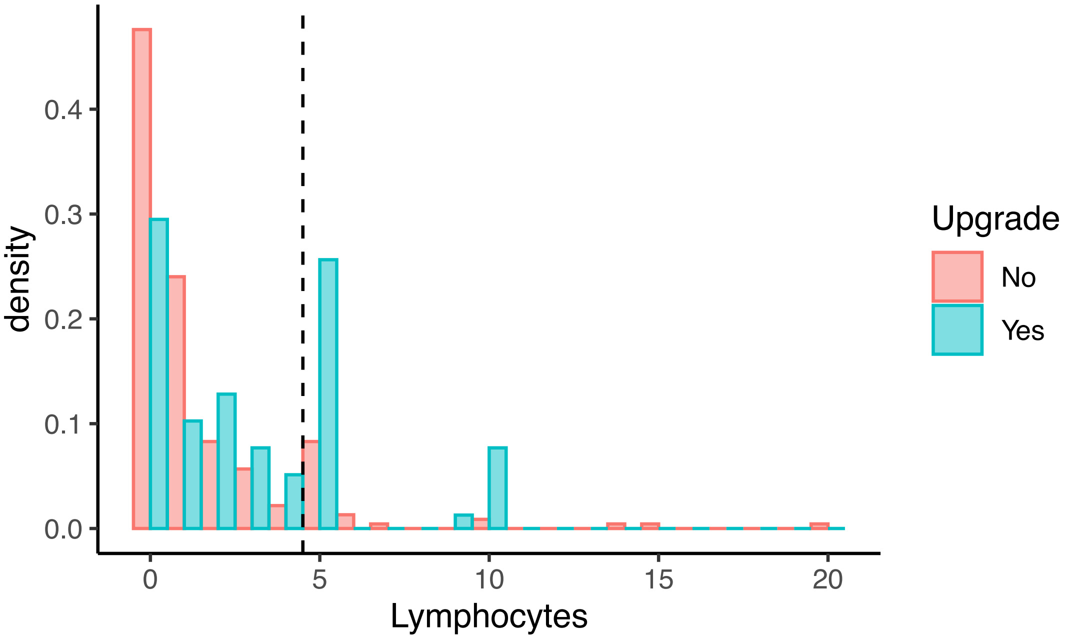

Supplement: Supplementary file 3 — Supplementary Material 3 [file 13058_2024_1857_MOESM3_ESM.pdf]
